# Walking orientation randomness metric (WORM) score: pilot study of a novel gait parameter to assess walking stability and discriminate fallers from non-fallers using wearable sensors

**DOI:** 10.1186/s12891-022-05211-1

**Published:** 2022-03-29

**Authors:** Ralph Jasper Mobbs, Pragadesh Natarajan, R. Dineth Fonseka, Callum Betteridge, Daniel Ho, Redmond Mobbs, Luke Sy, Monish Maharaj

**Affiliations:** 1 Faculty of Medicine, University of New South Wales, Sydney, Australia; 2 NeuroSpine Surgery Research Group (NSURG), Sydney, Australia; 3 Wearables and Gait Analysis Research Group (WAGAR), Sydney, Australia; 4 Department of Neurosurgery, Prince of Wales Hospital, Sydney, Australia; 5 Neuro Spine Clinic, Prince of Wales Private Hospital, 320-346 Barker St, Randwick, NSW 2031 Australia; 6 School of Mathematics and Computer Science, University of New South Wales (UNSW), Sydney, Australia

**Keywords:** Musculoskeletal disease, Gait disorders, Falls, Wearable device, Sensors, Gait analysis, Truncal motion

## Abstract

**Background:**

Musculoskeletal disorders can contribute to injurious falls and incur significant societal and healthcare burdens. Identification of fallers from non-fallers through wearable-based gait analysis can facilitate timely intervention to assist mobility and prevent falls whilst improving care and attention for high fall-risk patients. In this study, we use wearable sensor-based gait analysis to introduce a novel variable to assess walking stability in fallers and non-fallers – the Walking Orientation Randomness Metric. The WORM score quantifies the stability, or ‘figure-of-eight’ motion of a subject’s trunk during walking as an indicator of a falls-predictive (pathological) gait.

**Methods:**

WORM is calculated as the ‘figure-of-eight’ oscillation mapped out in the transverse-plane by the upper body’s centre-point during a walking bout. A sample of patients presenting to the Prince of Wales Hospital (Sydney, Australia) with a primary diagnosis of “falls for investigation” and age-matched healthy controls (non-fallers) from the community were recruited. Participants were fitted at the sternal angle with the wearable accelerometer, *MetaMotionC* (Mbientlab Inc., USA) and walked unobserved (at self-selected pace) for 5-50 m along an obstacle-free, carpeted hospital corridor.

**Results:**

Participants comprised of 16 fallers (mean age: 70 + 17) and 16 non-fallers (mean age: 70 + 9) based on a recent fall(s) history. The (median) WORM score was 17-fold higher (*p* < 0.001) in fallers (3.64 cm) compared to non-fallers (0.21 cm). ROC curve analyses demonstrate WORM can discriminate fallers from non-fallers (AUC = 0.97). Diagnostic analyses (cut-off > 0.51 cm) show high sensitivity (88%) and specificity (94%).

**Conclusion:**

In this pilot study we have introduced the WORM score, demonstrating its discriminative performance in a preliminary sample size of 16 fallers. WORM is a novel gait metric assessing walking stability as measured by truncal way during ambulation and shows promise for objective and clinical evaluation of fallers.

**Supplementary Information:**

The online version contains supplementary material available at 10.1186/s12891-022-05211-1.

## Background

Although quantification is difficult, changes in gait patterns are well-established and recognised in a variety of pathological conditions [1–4]. Traditionally gait analyses are performed in two distinct settings, each with its own benefits and disadvantages. The first is the *laboratory setting* which is typically objective with high accuracy but may be limited by expense, time consumption, and specialised material [5–8]. Further, with such synthetic testing conditions there is speculation that patterns may not be reflective of true everyday functions. In contrast the *clinical setting* (health care professional observation) may allow the observer to match one of several pathognomonic gait patterns, such as a Trendelenburg gait [9, 10], or neurogenic claudicant gait [11] to specific pathologies but may be limited by the degree of finite data able to be collected and the time for assessing the subject.

Inertial measurement units (IMU’s), commonly known as ‘wearable devices’ (*wearables*), contain various microelectromechanical sensors (MEMS) including accelerometers, gyroscopes and magnetometers. They have recently appeared as an alternative to the existing methods of gait assessment in the clinical setting [12, 13]. They are small, cheap, and marry the convenience of clinical assessment with the accuracy and objectivity of laboratory gait assessment. Additionally, unlike both existing methods, *wearables* provide the ability to observe walking in the absence of an observer, such as a clinician, eliminating the presence of any ‘white coat effect’ in a laboratory or when studied by a clinician [14]. Greater conscious control of walking can result in a representation of ‘best performance’ when observed (rather than ‘free-living’ gait) with overestimated cadence and underestimated gait variability [15].

Current commercial wearables can accurately measure numerous gait metrics including gait velocity, stride length, stride time, cadence, and step count [12]. The latest generation of devices are now able to detect more nuanced features of the gait cycle including aberrant (variable or asymmetric) movements, exaggerated axial sway or range of joint motion [13]. Despite this, integration to the health setting has been limited, though potential utility is great. In addition to assessing disability and post-intervention recovery, wearable devices may also be useful in the identification of falls-risk patients [16].

Falls incur a significant disease burden annually, resulting in 695,771 deaths and a vast 35,940,787 disability-adjusted life years (DALYs) lost globally during 2017 [17]. Current falls-risk stratification strategies in clinical settings rely on structured questionnaires based on well-established risk factors including falls history, sedative medications, altered psychological states and gait or balance disorders. However, clinical accuracy in identifying patients at high risk of falls has been found to be limited [18], with Chapman et al’s (2011) assessment of four common tools (including Morse Fall Scale and Hendrich II Fall Risk Model) demonstrating sensitivity to range from 57.1–100% and specificity from 24.9–69.3% [19]. There is a clear need to implement more accurate falls prediction and prevention strategies, particularly leveraging recent wearable sensor capabilities.

Falls may be caused by an interplay between (patient-related) intrinsic and (environmental) extrinsic risk factors [20–23]. Despite multiple causes, balance and gait abnormalities have been consistently identified in the literature as one of the primary causes [21, 24–26]. Objective studies of fallers’ gait has demonstrated spatiotemporal alterations, with some of these variables such as gait velocity, cadence and stride length further explored for falls-classification [27, 28]. One hypothesis is that balance dysfunction and poor postural control account for inconsistent stepping patterns and therefore drive a greater gait variability [29, 30]. As a consequence the stride-to-stride variability of gait parameters have also been found to be useful [31], despite differing measures of standard deviation [32] or coefficient of variation being used to examine fall related gait behaviour [33, 34].

Previous wearable accelerometry studies of walking stability in fallers have assessed the smoothness and rhythm (variability) of acceleration patterns with the Lyapunov exponent [35, 36], autocorrelation coefficient [34, 37] and harmonic ratio [34, 38, 39]. Previous authors have typically considered balance in the mediolateral, vertical, and anteroposterior planes [34, 39–41]. Trunk-based sensor-placement has been widely employed as it is most proximal to the centre of mass (COM) [33, 41, 42]. Whilst most studies employed placement at the lumbar vertebrae [33, 39, 42], some have used chest-based sensor placement [41, 43].

The COM is calculated to lie a few centimetres anterior to the lumbosacral joint [44], with most wearable sensor-based studies analysing COM motion opting for sensor-placement along the lumbar vertebrae [33, 39, 42]. However, these lumbosacral approximations do not entirely reflect the movement of each individual body segment of ambulation (especially the upper limbs) which can largely influence COM motion and walking stability [45, 46]. We hypothesise that sensor placement in the midline (anterior) chest wall captures truncal and limb motion, providing a holistic measure of walking stability.

Indeed, chest-based sensor placement has been validated in the literature to provide reliable and accurate measurement of postural [41, 47, 48] and spatiotemporal gait parameters [43, 49]. Chest-based sensor placement has also been previously used to measure truncal sway and assess ataxic gait [50, 51]. Previous studies have demonstrated fallers experience greater displacements and velocities of truncal sway in the anteroposterior and mediolateral planes [52]. However, an integrative approach combining accelerometric, gyroscopic and magnetometry inputs to measure movement of the trunk’s centre-point in the transverse plane (i.e path lengths) has previously not been explored.

This technical description therefore aims to provide a standardised unit to assess walking stability and discriminate between fallers and non-fallers. Using a chest-based wearable device, we introduce a novel variable that quantifies the transverse-plane motion of a subject’s trunk during walking – hereby referred to as the Walking Orientation Randomness Metric (WORM).

## Methods

### Walking orientation randomness metric (WORM)

During walking, the summative motion of individual joint segments accelerates the trunk forwards of the base of support. With each step, the trunk also oscillates side-to-side (about 46 mm), over each leg during its stance phase [53, 54]. Subtracting the average forward velocity of the body, shows the subjects’ trunk to therefore oscillate rhythmically left-right [45]. This lateral truncal displacement is pertinent from a clinical standpoint as lateral stability is ultimately compromised in many neurological and orthopaedic pathologies [55].

**Fig. 1:**
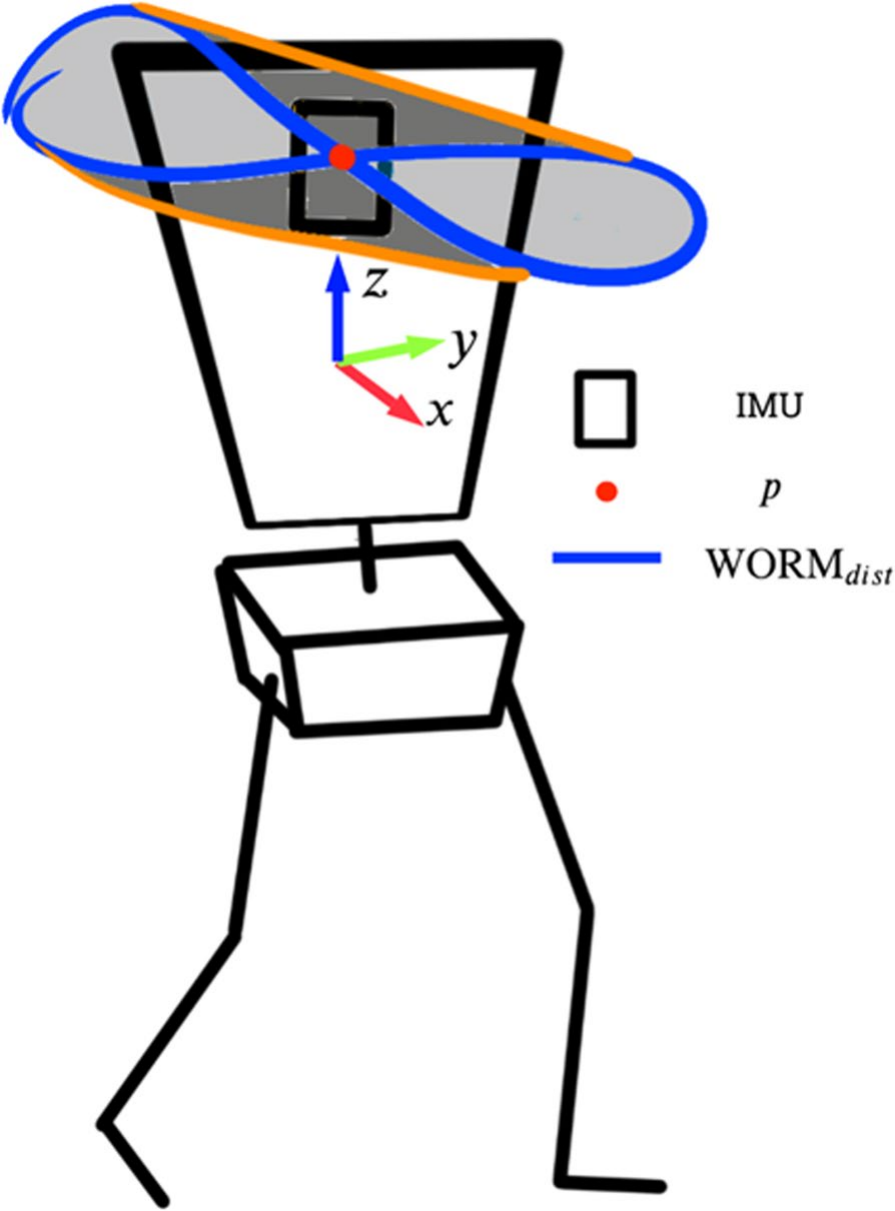
Diagrammatic model of human body during walking. WORM score calculation reflects truncal motion as a measure of walking (in)stability. WORM_dist_ is calculated as the distance travelled in the transverse plane travel by p, (the length of the blue outline) measuring truncal motion during the walking bout. IMU = Inertial Measurement Unit, WORM = Walking Orientation Randomness Metric

To assess the stability of this inverted pendulum-like motion during walking [56–58], the WORM score measures the ‘figure-of-eight’ motion of the upper body derived from a chest-based wearable device as shown in Fig. 1*.* The single-point IMU provides real-time quaternions that are subsequently converted to Euler angles. These three-dimensional angles are then used to calculate the trajectory of the subject’s truncal sway (Eq. 1–3). We first calculate the point p_t_ at time step t from the orientation of the body with respect the world frame, ${{{}{}^{\mathrm{W}}\mathrm{R}}_{\mathrm{t}}^{\mathrm{B}}}$. The body orientation, ${{}{}^{\mathrm{W}}\mathrm{R}}^{\mathrm{B}}$, is obtained from the orientation measured by the single point IMU, ${{}{}^{\mathrm{W}}\overset{\smile }{\mathrm{R}}}_{\mathrm{t}}^{\mathrm{S}}$, adjusted by a fixed sensor-to-body rotational offset, ${{}{}^{\mathrm{B}}\mathrm{R}}_0^{\mathrm{S}}$, as shown in Eq. 1. The sensor-to-body offset, ${{}{}^{\mathrm{B}}\mathrm{R}}_0^{\mathrm{S}}$, was calculated by assuming an upright pose (i.e., ${{}{}^{\mathrm{W}}\mathrm{R}}_0^{\mathrm{B}}={\mathrm{I}}_{3\mathrm{x}3}$) at t = 0 as shown in Eq. 2. Finally, point p_t_, which is effectively the x and y coordinates of the body z axis with centre at the origin, is calculated using Eq. 3 (the three-dimensional body orientation is projected in two-dimensions, the transverse plane). From point p_t_, WORM_dist_ is calculated as the distance travelled in the transverse plane travel by p_t_, (the length of the blue outline) measuring truncal motion during the walking bout (Fig. 1).

1 $${{}{}^{\mathrm{W}}\mathrm{R}}_{\mathrm{t}}^{\mathrm{B}}={{}{}^{\mathrm{W}}\overset{\smile }{\mathrm{R}}}_{\mathrm{t}}^{\mathrm{S}}{\left({{}{}^{\mathrm{B}}\mathrm{R}}_0^{\mathrm{S}}\right)}^{\mathrm{t}}$$

2 $${{}{}^{\mathrm{B}}\mathrm{R}}_0^{\mathrm{S}}={{}{}^{\mathrm{W}}\overset{\smile }{\mathrm{R}}}_0^{\mathrm{S}}$$

3 $${\mathrm{p}}_{\mathrm{t}}=\left[\begin{array}{ccc}1& 0& 0\\ {}0& 1& 0\end{array}\right]{{}{}^{\mathrm{W}}\mathrm{R}}_{\mathrm{t}}^{\mathrm{B}}\left[\begin{array}{c}0\\ {}0\\ {}1\end{array}\right]$$

This WORM_dist_ output is subsequently averaged to time walked and to distance walked to derive the final WORM Score (hereafter referred to simply as WORM). Thus, WORM measures the “figure-of-eight” motion of the trunk in the transverse plane, averaged per metre and per second of walking.

### Study participants

The participants of this study were a sample of patients presenting to the Prince of Wales Hospital (Sydney, Australia) with a primary diagnosis of “falls for investigation” in July–August 2020. During their acute hospital admission, study parameters and risks were discussed, and consent obtained. Participants lacking the ability to walk any distance without a form of support (walking stick or frame) were excluded. Participants with any form of orthopaedic injury following their fall (for instance neck of femur fracture), that may exacerbate their gait dysfunction were also excluded. Included patients underwent a semi-structured interview to obtain demographic information and assess eligibility. To be included in the “fallers” group, the fall must have been unrelated to a medication event and the patient must have intact binocular vision without concurrent visual pathologies. Age-matched “non-faller” controls were recruited from the community following a similar semi-structured interview. Ethics for this study was obtained from the South Eastern Sydney Local Health District, with reference code 17/184.

### Study design

A retrospective study design was used to group fallers, based on recent history of falls (in the week of community ambulation preceding admission). A retrospective study design was opted for this pilot study due to the ethical concerns and technical difficulties of prospective long term community follow-up of ‘potential fallers’. Moreover, categorisation of faller status based on recent history of falls is reasonably justified by the fact that history of previous falls strongly predicts future falls [20, 21, 59]. The authors of the study believed that, assuming no major injuries had been sustained from the high fall population that their inpatient gait patterns were reasonably reflective of their pre-fall pattern of ambulation. Controls were included to the non-fallers group, based on no previous history of falls and age-matching with participants of the fallers group.

### Sample size calculations

Required sample size of 14 participants per group was calculated using the GPower 3.0 program to achieve at least 80% power given an effect size of 1. A recruitment target of at least 15 participants was therefore set for this preliminary study to account for any potential data losses.

### Procedure

Prior to the walk, participants were fitted at the sternal angle (Supplementary Materials - Appendix B) with an inertial measurement unit: the MetaMotion© (MMC) manufactured by Mbientlab Inc. (California, USA). In addition, patients wore a safety belt such that any fall during the subsequent walking event could be prevented by the 3 investigators who were in close proximity to the patient walking for safety. Following a short initial pause to orient the MMC device, participants walked a self-selected distance (5–50 m) with (mean +/− standard deviation) distance walked by fallers being 17 +/− 18 m whilst non-fallers walked 49 +/− 2.8 m. Participants walked as far as they could consent to walk safely, at a self-selected pace along a 50 m unobstructed pathway on level ground. Trials were discarded if the patient did (or could) not pause to orient the device, walk more than 5 m or required walking assistance during the bout.

### Data processing

The MMC is a wearable sensor that involves data fusion (using a Kalman filter) from a 16 bit 100 Hz triaxial accelerometer, a 16 bit, 100 Hz triaxial gyroscope and a 0.3 μT, 25 Hz triaxial magnetometer. Captured data is stored as a matrix of the values corresponding to each time point (100 captures per second) for up to 20 min of walking. For the purposes of this study, the MMC device recorded the entire walking trial, and the data captured was transmitted via Bluetooth™ to an Android™ smartphone running the IMUGait Recorder application developed for this study (Supplementary Materials - Appendix B). The IMUGait Recorder application then uploaded the raw data to a centralised database where a modified version of Czech et al’s open-source python program (IMUGaitPy program) was used to process the gait metrics for that walking trial [60], and visualise the WORM Score (Supplementary Materials - Appendix C). The MMC was used to measure truncal sway, and IMUGaitPy program was then used to calculate the Walking Orientation Randomness Metric (WORM) score.

### Statistical analysis

Data analyses were performed using Prism 9 (GraphPad Software). Descriptive statistics were calculated for demographic variables including; age, gender, presence of diabetes and smoking. Spatiotemporal parameters of gait were calculated, and step (rather than stride) measurements chosen for calculations of asymmetry and variation due to greater reliability [61]. Differences between fallers and non-fallers were calculated using unpaired two-tailed t-test. Welch’s correction was applied for variables with unequal variance and Mann Whitney U test used where non-normal distribution was present. Discriminative ability was assessed using the area under the curve values of receiver operating characteristic curves for each gait metric. Accuracy values were interpreted as follows: 0.5 = test due to chance, 0.7–0.9 = moderate accuracy, 0.9–1.0 = very accurate, 1.0 = perfect test. Normality was assessed using Shapiro-Wilk tests and inspection of histograms. Statistical significance was considered with a *p*-value < 0.05.

## Results

### Participant demographics

16 participants recruited as ‘fallers’ had a range of comorbidities including delirium, hip/knee osteoarthritis, lumbar radiculopathy, scoliosis, vestibular imbalance, cervical myelopathy, foot-drop and stroke-related hemi-paresis. The 16 control participants recruited as ‘non-fallers’ also had various comorbidities consistent with their age such as osteoarthritis of the spine and lower extremities, however had no history of falls. Demographic variables including age, gender ratios, smoking and diabetic status, body mass indices and height for these participants were not significantly different between fallers and non-fallers, with the exception of weight and daily step count (Table 1).

**Table 1:** Demographic and clinical characteristics of fallers and non-fallers. *P* value represents statistical significance of difference between groups derived from Unpaired two-tailed t-test (Welch’s correction* applied if unequal variance), Mann Whitney U tests** (if non-normal distribution) or Fisher’s Exact Test^†^. Significant findings are bolded

	Fallers	Non-Fallers	P
N	16	16	n/a
Age (mean + SD, years)	70 + 17	70 + 9	0.609*
Gender ‘M’ (%)	8 (50)	10 (62.5)	0.722^†^
Height (mean + SD, cm)	168 + 10	174 + 11	0.154
Weight (mean + SD, kg)	73 + 19	88 + 21	**0.042**
BMI (mean + SD, kg/m^2^)	26 + 5.6	29 + 5.6	0.099
Smoking (%)	1 (6.25)	0 (0)	n/a
Diabetes (%)	0 (0)	2 (12.5)	n/a
Daily Step Count (mean +/− SD, steps)	650 + 540	4420 + 4050	< 0.001**

### Spatiotemporal gait parameters

9 gait characteristics were measured across the four main gait domains of spatial, rhythm/temporal, asymmetry and variation metrics. 8 of these were significantly different between fallers and non-fallers (Table 2), and 7 of these remain significant following Bonferroni’s corrections (*p* = 0.05/13 = 0.0038) for multiple testing. Fallers have a typical gait pattern of significantly lower gait velocity (− 43%), step length (− 27%) and cadence (− 23%) significantly increased parameters include step time (+ 37%), step time asymmetry (+ 305%) and variability in terms of gait velocity (+ 114%), step time (+ 150%) and step length (+ 128%). Asymmetry in step length was not found to be significantly different (*p* = 0.080) in fallers.

**Table 2:** Differences in gait parameters between Fallers and Non-Fallers. P value represents statistical significance of difference between groups derived from Unpaired two-tailed t-test, Welch’s corrected t-tests*, or Mann Whitney U tests**. WORM calculated as average distance (cm) per metre (mean/m) and per second (mean/s) of walking

	Fallers	Non-Fallers	Between Group Differences (Fallers – Non-Fallers)			
	Mean + SD		Mean / Median Difference	95% Confidence Interval	Difference (%)	P
**Spatial Gait Metrics**						
Gait Velocity (m/s)	0.637 + 0.261	1.13 + 0.252	−0.489	−0.674 to − 0.304	−43%	**< 0.001**
Step Length (cm)	45.3 + 13.6	62.0 + 13.7	−16.8	−26.6 to −6.90	−27%	**0.002**
**Rhythm/Temporal Gait Metrics**						
Cadence (steps/min)	84.5 + 14.3	109 + 7.57	−24.8	−33.2 to −16.5	−23%	< 0.001*
Step Time (ms)	757 + 139	554 + 38.7	203	127 to 279	+ 37%	< 0.001*
**Gait Asymmetry**						
Step Time Asymmetry (ms)	191 + 108	47.1 + 26.6	144	85.3 to 202	+ 305%	< 0.001*
Step Length Asymmetry (cm)	13.4 + 8.65	8.10 + 3.42	2.8	−0.304 to 7.23	+ 65%	0.080**
**Gait Variability**						
Gait Velocity Variation (CV%)	15.2 + 7.01	7.09 + 2.17	8.08	4.09 to 12.1	+ 114%	**0.001**
Step Time Variation (CV%)	18.9 + 8.63	7.53 + 3.71	11.3	6.26 to 16.4	+ 150%	**0.001**
Step Length Variation (CV%)	23.9 + 14.6	10.5 + 4.52	13.4	5.11 to 21.7	+ 128%	**0.003**
**Gait Stability**						
WORM	3.64 ± 3.90	0.211 ± 0.171	2.49	1.01 to 3.61	+ 1179%	< 0.001**

*CV%* Coefficient of Variance* 100, *WORM* Walking Orientation Randomness Metric

The ability of these spatiotemporal parameters of gait to differentiate between fallers and non-fallers was assessed by statistically significant area under the curve (AUC) values of receiver operating characteristic (ROC) curves (Table 3). Good accuracy was found for most gait parameters with the highest accuracy found in gait velocity (AUC = 0.91), step time (AUC = 0.94), asymmetry in step time (AUC = 0.90), and variation in gait velocity (AUC = 0.90).

**Table 3:** AUC values of ROC curves for gait parameters in discriminating between Fallers and Non-Fallers

	AUC	Std. Error	95% Confidence Interval		P
			Upper Bound	Lower Bound	
**Spatial Gait Metrics**					
Gait Velocity (m/s)	**0.910**	0.051	1.000	0.810	< 0.001
Step Length (cm)	0.801	0.079	0.955	0.647	0.004
**Rhythm/Temporal Gait Metrics**					
Step Time (ms)	**0.938**	0.045	1.000	0.849	< 0.001
**Gait Asymmetry**					
Step Time Asymmetry (ms)	**0.902**	0.060	1.000	0.787	0.001
Step Length Asymmetry (cm)	0.684	0.095	0.870	0.497	0.077
**Gait Variability**					
Gait Velocity Variation (CV%)	**0.904**	0.056	1.000	0.794	0.001
Step Time Variation (CV%)	0.875	0.068	1.000	0.741	0.001
Step Length Variation (CV%)	0.825	0.079	0.981	0.670	0.002
**Gait Stability**					
WORM	**0.965**	0.028	1.000	0.9108	< 0.001

*AUC* Area under the curve*, ROC* Receiver Operating Characteristic*, Std. Error Standard Error, CV%* Coefficient of variance* 100, *WORM* Walking Orientation Randomness Metric

### WORM scores

Walking stability according to WORM scores (cm/s) was significantly different (*p* < 0.001), being 17-fold higher (mean ± standard deviation) in fallers (3.64 ± 3.90) compared to non-fallers (0.21 ± 0.17). These differences in WORM scores (as seen in Tables 2 and 3) show high accuracy (AUC = 0.97) in differentiating fallers from non-fallers with a sensitivity of 87.50% and specificity of 93.75% when selecting the cut-off (WORM > 0.51 cm) with highest likelihood ratio (14.00).

## Discussion

Through wearable accelerometry we have identified the relevant gait variables with high discriminative power in classifying fallers from non-fallers: gait velocity, step time, gait asymmetry (in step time) and gait variability (in gait velocity). Our identification of significantly different gait parameters in fallers largely aligns with existing findings in literature such as reduced walking speed [27, 28, 62], cadence [28, 62] and stride/step length [27, 62] with greater step time (in double support) [28], gait variability (in swing time) [28] and gait asymmetry [39, 62]. These gait deficits in the fallers group could be attributable to dysfunction of muscle strength, balance, proprioceptive physiology and/or disuse-related [63]. In this pilot study we have also introduced the WORM score, demonstrating its discriminative performance in a preliminary sample size of 16 fallers, affirming clinical utility for further research.

The novel gait metric investigated in the present study (WORM), assesses walking stability as measured by truncal motion in the transverse-plane during walking. The rationale for WORM stems from existing theories surrounding centre of mass (COM) motion [64–66] during ambulation, when considering the translation of the body system as a whole. Previous clinical studies and mathematical modelling suggests COM motion to undergo a closed figure-of-eight path (a ‘bow-tie’ shape), upwardly concaved in the frontal plane [65, 67–69]. Measurement of COM trajectory (especially its lateral motion) is thus clinically relevant to understanding and predicting falls, as they mostly occur towards the lateral direction [55].

WORM’s methodology provides an alternative to measuring ‘COM’ at the lower lumbar vertebrae. We believe a chest-based sensor placement incorporates compensatory truncal inclinations and/or upper limb motions (that seek to offset pathological lower limb biomechanics) in assessing walking stability. Greater truncal motion due to these compensatory gait alterations likely enabled discrimination of fallers from non-fallers via WORM in the present study. Our finding aligns with consensus in literature regarding truncal sway measurements offering useful information to distinguish gait abnormalities and fallers [70, 71].

The WORM calculation (averaged as mean per metre and per second of walking) was justified by Fukuchi et al’s recommendations when analysing pathological gait patterns, to consider the confounding effects of higher gait speeds increasing the amplitudes of spatiotemporal parameters, joint kinematics, joint kinetics, and ground reaction forces [72]. This is likely due to velocity-related changes in the total length of the figure-of-eight path due to shortening of its lateral oscillations [45]. As manifested in the present study, the relationship between truncal sway and gait velocity in older adults offers insight into differences between fallers and non-fallers [70]. Although truncal sway decreases with increased gait velocity in clinically normal gait according to Tesio et al. 2019 [45], this relationship likely ceases to exist in pathological gait thereby accounting for greater WORM scores in fallers.

The proposed method presents an objective, unsupervised and unobtrusive method of point-of-care testing to assess walking stability and balance in both clinical and community settings. The WORM score provides clinicians, patients and carers with a quantification of walking instability serving as an accurate and sensitive biomarker for falls-risk. WORM may guide falls-preventative interventions in the elderly such as mobility assistance (walking aids), home modifications [73], alterations to medication regiments [74, 75], or physical therapy [76]. We have reported WORM scores for non-fallers versus fallers, however the intermediate scores between these 2 points may provide further insight into fall-risk stratification to guide these interventions. WORM may also serve clinical utility in minimising post-intervention falls in the community for example when planning safe discharge, rehabilitation and home care [77].

A limitation of the present study would be the classification of faller status based on retrospective history (albeit recent in the preceding week). Although, classification of faller status based on retrospective history can be justified by the fact prior history of falls strongly predicts future falls [20, 21, 59], future studies in prospective fallers may enable confirmation of ‘real-world’ falls-prediction capabilities in community and at-home settings. Despite promising findings in this preliminary study, the sample size of 32 participants is a limitation. Moreover, accuracy and re-test reliability is unknown without future validation in larger external datasets. Moreover, challenges commonly faced by medical wearable devices such as battery life and missed communication (sensor failure and consequent data loss for one patient), were also experienced in the present study [78].

We have reported on a single aspect of walking instability (velocity of truncal sway) being sensitive and specific in our sample population of fallers. However, future research may consider the utility of path lengths and area of truncal sway. The discriminative performance of these WORM scores may vary among pathologies. Other avenues of research include: defining normative values of WORM depending on age and sex, leveraging artificial intelligence classification techniques (such as machine learning, deep neural learning) in future analyses and validation in more specific pathological populations (knee/hip osteoarthritis, multiple sclerosis, Parkinson’s disease).

## Conclusion

In this pilot study we have also introduced the WORM score, demonstrating its discriminative performance in distinguishing high-risk falls patients from an age-matched cohort of non-fallers. WORM is a novel gait metric assessing walking stability as measured by truncal motion during ambulation and shows promise for falls prediction.

## Supplementary Information

**Additional file 1.**

## Data Availability

The datasets generated during and analysed during the current study are not publicly available due to authors’ ownership of intellectual property rights but are available from the corresponding author on reasonable request.
