# Supplementary material for: Walking orientation randomness metric (WORM) score: pilot study of a novel gait parameter to assess walking stability and discriminate fallers from non-fallers using wearable sensors
Source: BMC Musculoskelet Disord. 2022 Mar 29;23:304. doi: 10.1186/s12891-022-05211-1 (PMC8966274; doi:10.1186/s12891-022-05211-1)
Supplement: Supplementary file 1 — Additional file 1. [file 12891_2022_5211_MOESM1_ESM.docx]

***
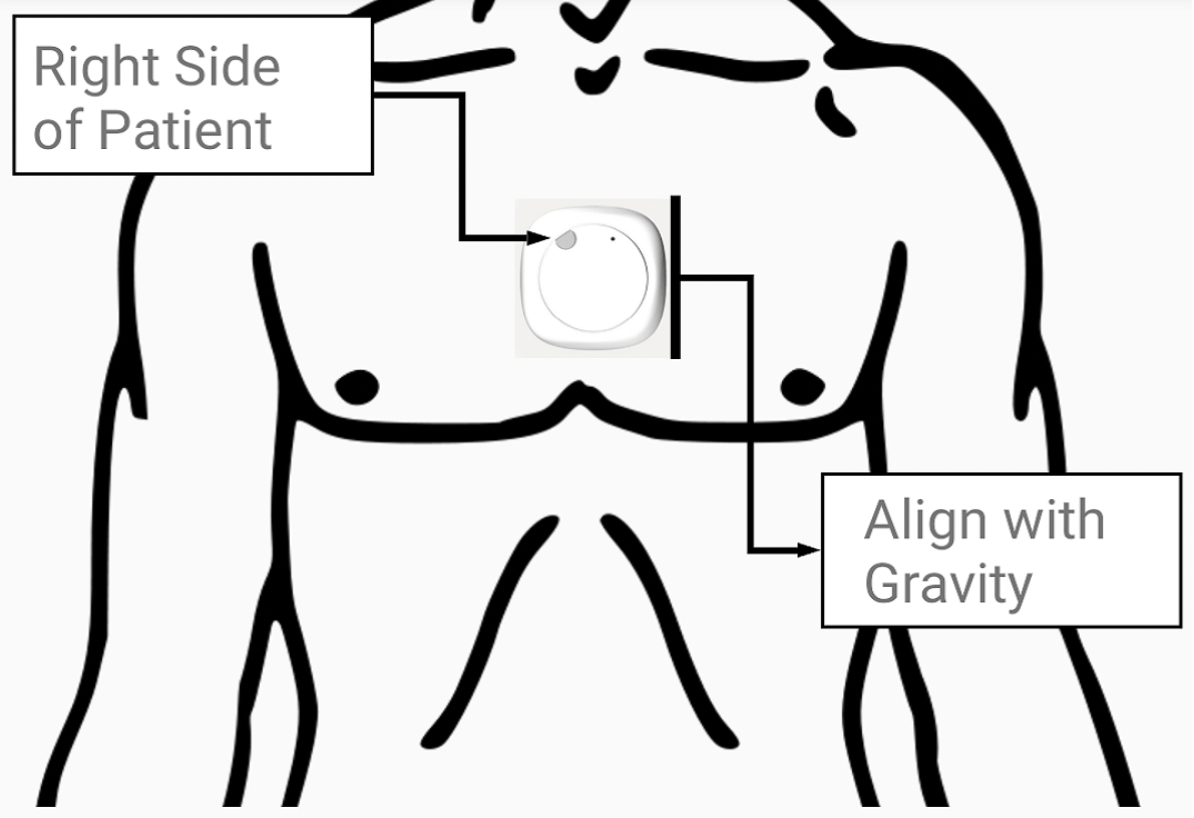
***

***Appendix A*. Frontal view of subject showing position of MetaMotionC wearable device attachment, prior to walking episode.** **Reproduced with the permission of Betteridge et al^1^**.

Device was placed on the skin immediately superior to the sternal angle for gait analysis of both fallers and non-fallers. A chest-based sensor placement in the midline anterior chest wall is likely represents the most holistic measure of walking (in)stability, capturing (pathological) truncal and upper limb motions.


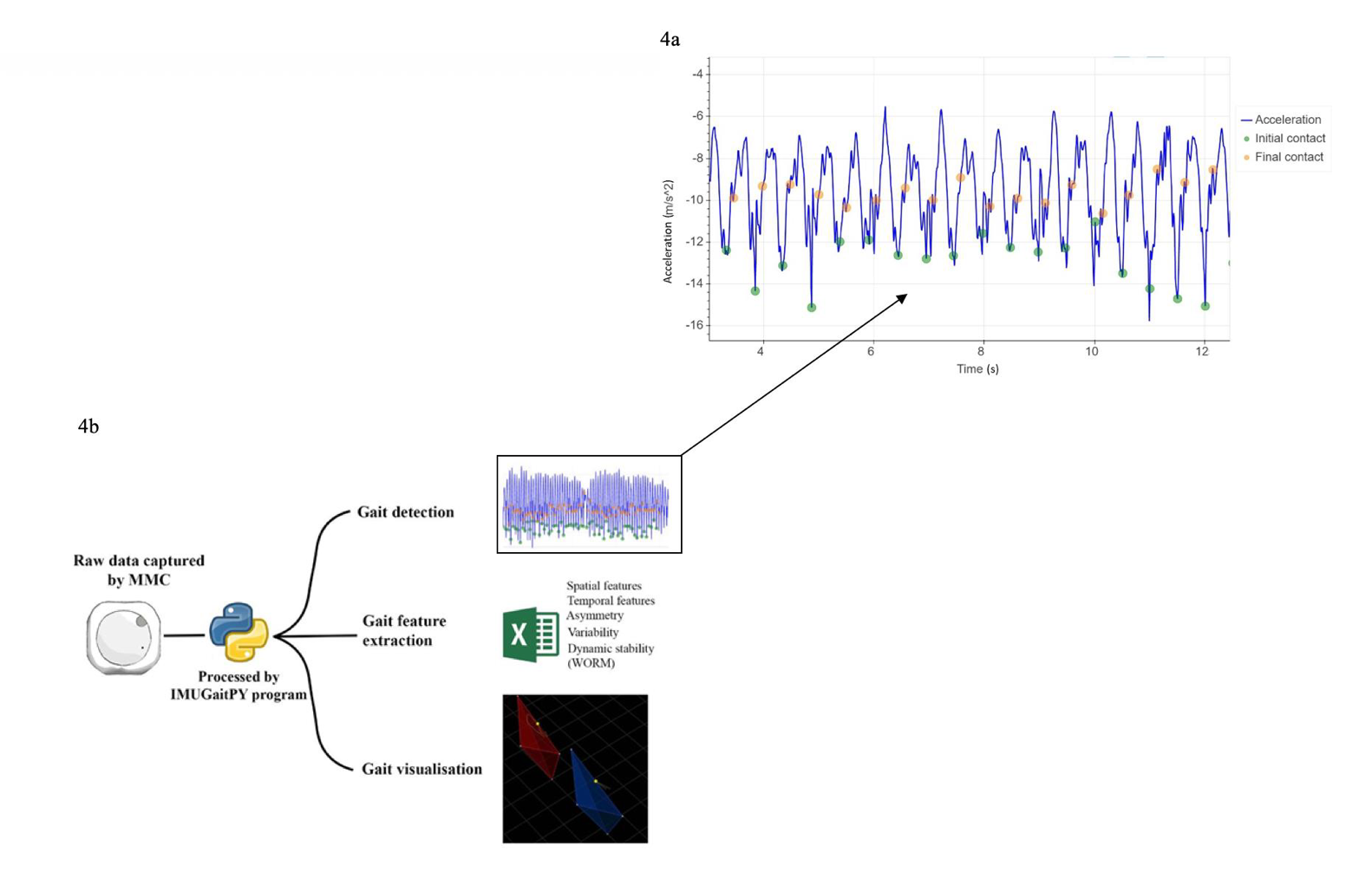
***Appendix B*. Summary of data collection, processing, and outputs from the MetaMotionC sensor and IMUGaitPY program for gait analysis used in this study. Reproduced with the permission of Betteridge et al^1^**.

*i*. First output is a .html file which documents the vertical acceleration measured by the sensor (y-axis) against time (x-axis) during the walk done by the participant. Green circles represent the initial foot contact with the ground, usually the ‘heel strike’ phase of gait and orange circles represent the final foot contact with the ground, usually the ‘toe-off’ phase of gait.

*ii*. The IMUGaitPY program uses the gait cycle events detected in image *a* to identify when gait cycles begin and end, and thus creates a .csv file with the values of each gait parameter displayed per gait cycle and for the bout overall. For calculations see *Appendix A*. Additionally, a .c3d file is created which can be viewed using Mokka, an open source platform, and the configuration file in Appendix A. This creates a visual recreation of the gait using the accelerometry data.

WORM = walking orientation randomness metric, MMC = MetaMotionC sensor from Mbientlabs, used to measure gait in the present study.


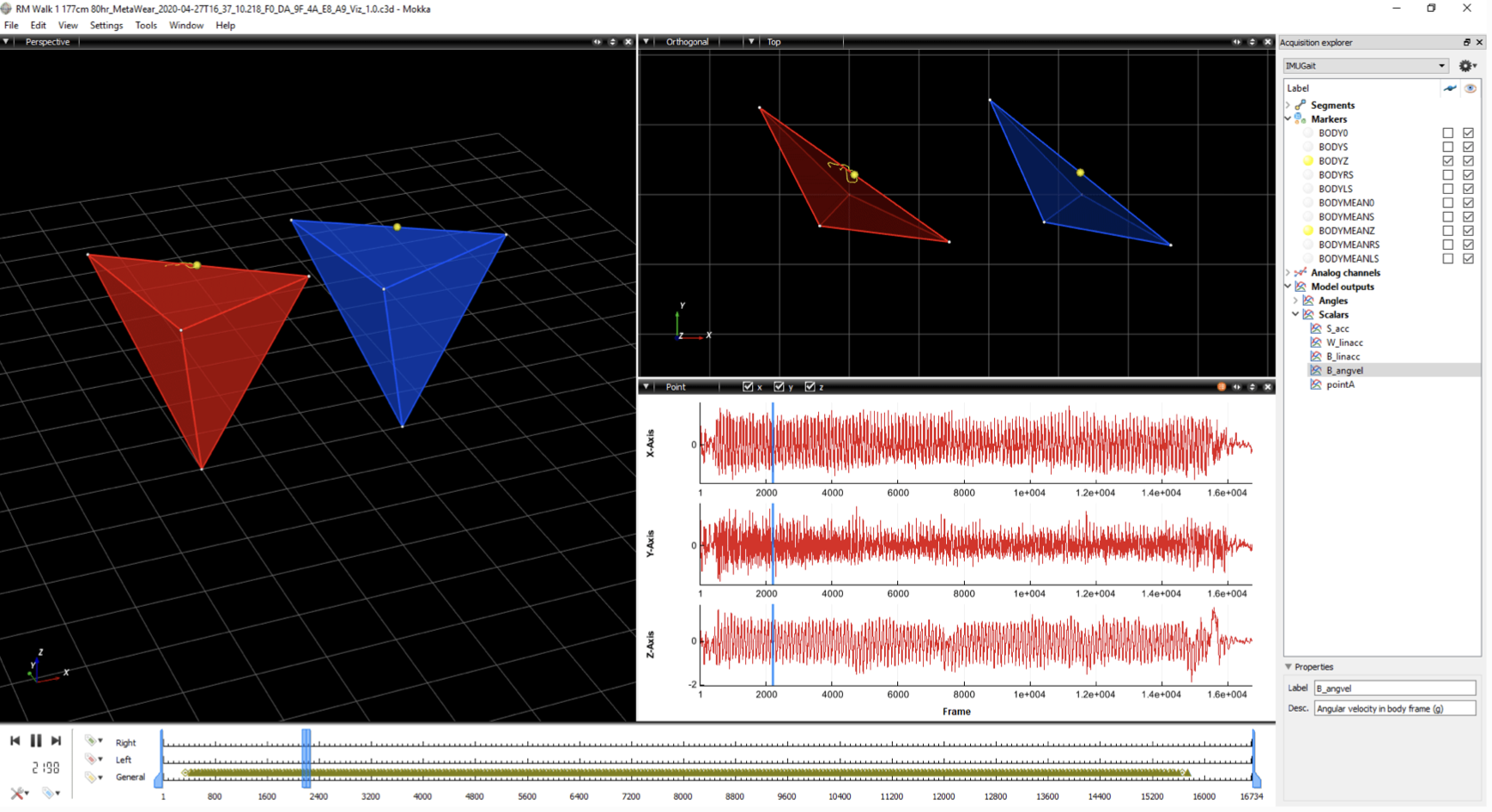


***Appendix C*. Visualisation of WORM Score.** *Mokka software* is used to create visual recreation of gait stability using the inertial measurement unit data for an example participant..

1 Betteridge, C. et al. Objectifying clinical gait assessment: using a single-point wearable sensor to quantify the spatiotemporal gait metrics of people with lumbar spinal stenosis. *Journal of Spine Surgery* **7**, 254 - 268, doi:<https://dx.doi.org/10.21037/jss-21-16> (2021).
